# Biologically important artificial light at night on the seafloor

**DOI:** 10.1038/s41598-020-69461-6

**Published:** 2020-07-27

**Authors:** Thomas W. Davies, David McKee, James Fishwick, Svenja Tidau, Tim Smyth

**Affiliations:** 1 School of Biological and Marine Sciences, University of Plymouth, Drake Circus, Plymouth, PL4 8AA UK; 2 School of Ocean Sciences, Bangor University, Menai Bridge, Anglesey, LL59 5AB UK; 3 Physics Department, University of Strathclyde, 107 Rottenrow, Glasgow, G4 0NG Scotland; 4 Plymouth Marine Laboratory, Prospect Place, Devon, Plymouth, PL1 3DH UK

**Keywords:** Physical oceanography, Marine biology, Urban ecology, Microbial biooceanography

## Abstract

Accelerating coastal development is increasing the exposure of marine ecosystems to nighttime light pollution, but is anthropogenic light reaching the seafloor in sufficient quantities to have ecological impacts? Using a combination of mapping, and radiative transfer modelling utilising in situ measurements of optical seawater properties, we quantified artificial light exposure at the sea surface, beneath the sea surface, and at the sea floor of an urbanised temperate estuary bordered by an LED lit city. Up to 76% of the three-dimensional seafloor area was exposed to biologically important light pollution. Exposure to green wavelengths was highest, while exposure to red wavelengths was nominal. We conclude that light pollution from coastal cities is likely having deleterious impacts on seafloor ecosystems which provide vital ecosystem services. A comprehensive understanding of these impacts is urgently needed.

## Introduction

The potential for artificial light at night (ALAN) to reshape the ecology of marine habitats is increasingly recognised, and an emergent focus of research^1–4^. Artificial light can be detected above 22% of the world’s coasts nightly^5^, and will dramatically increase as coastal human populations more than double by year 2060^6^.

Given the low levels of artificial light that likely reach the seafloor, it seems intuitive to suggest that light pollution is not a concern in marine ecosystems. Marine organisms are however, evolutionarily adapted for detecting natural light of low intensity, distinct spectra and regular cycles. To give some examples, *Calanus* copepods undergo diel vertical migration to depths of 50 m guided only by variations in moonlight intensity during the Arctic winter^7,8^; the larvae of some sessile invertebrates move and identify suitable settlement locations guided by light levels equivalent to moonless overcast nights^9^; and polychaete worms, corals and echinoderms synchronise broadcast spawning events using monthly and annual variations in lunar light intensity^10^. In-water radiative transfer modelling reveals that the larval and adult stages of zooplankton, tropical corals and temperate marine organisms are likely to respond to artificial sky glow (light scattered in the atmosphere and reflected back to the ground) down to depths of 70 m, and to waterside street lighting down to 100 m^11^. These predictions are corroborated in part by recent observations of zooplankton avoiding research vessel lights at depths > 80 m^1^. Given the high sensitivity of marine animals to light, and the extent of ALAN across coastal regions^5^, large areas of seafloor habitat adjacent to urbanised coastlines are likely experiencing light pollution levels that are detectable to marine organisms and, as a consequence, impacting marine ecosystems.

The growing use of white Light Emitting Diodes (LEDs)—forecast to account for up to 80% of the global lighting market share by 2022^12^—will likely exacerbate the prevalence and impacts of artificial light in marine ecosystems**.** Compared to older lighting technologies, LEDs emit more short wavelength light that: (i) penetrates deeper into seawater [the spectral signature from land being detectable on coral reefs at 30 m depth^13^]; and (ii) many marine organisms are most sensitive to^7,14^. Given the pace at which LEDs are being adopted in coastal cities around the world, an understanding of the prevalence of ‘biologically important’ artificial light pollution (irradiances sufficient to elicit responses in marine organisms) is urgently needed.

## Results

**Figure 1:**
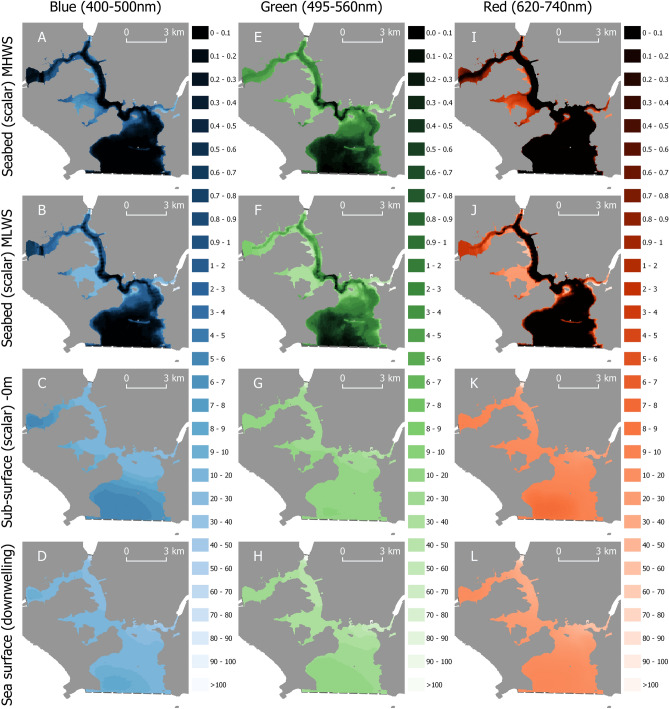
The spatial distribution of artificial light at night across Plymouth Sound and the Tamar Estuary, UK in cloudy conditions (5–8 Okta). Modelled scalar irradiances (µW m^−2^) are given for Blue (400-500 nm, **A**–**D**), Green (495–560 nm, **E**–**H**), and Red (620-740 nm, **I**–**L**) light on the seabed during Mean High Water Spring (MHWS) tide (**A**,**E**,**I**), Mean Low Water Spring (MLWS) tide (**B**,**F**,**J**), and immediately beneath the water surface (**C**,**G**,**K**). Measured planar irradiances are given for light incident on the sea surface (**D**,**H**,**L**). The coordinate reference system is OSGB 1936/British National Grid. Land is given by solid grey regions, the survey extent by dashed grey lines. Maps created in QGIS v3.4 (https://qgis.org).

**Figure 2:**
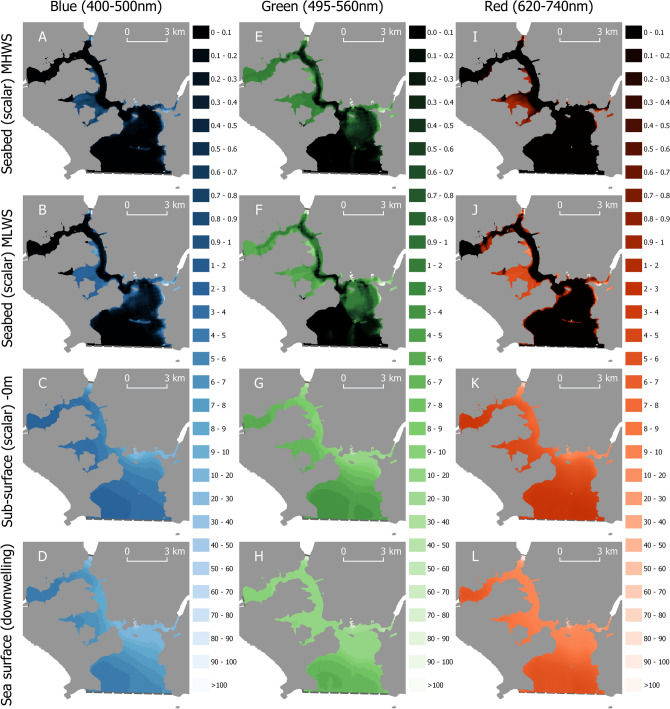
The spatial distribution of artificial light at night across Plymouth Sound and the Tamar Estuary, UK under clear conditions (0–3 Okta). Modelled scalar irradiances (µW m^−2^) are given for Blue (400–500 nm, **A**–**D**), Green (495–560 nm, **E**–**H**), and Red (620-740 nm, **I**–**L**) light on the seabed during Mean High Water Spring (MHWS) tide (**A**,**E**,**I**), Mean Low Water Spring (MLWS) tide (**B**,**F**,**J**), and immediately beneath the water surface (**C**,**G**,**K**). Measured planar irradiances are given for light incident on the sea surface (**D**,**H**,**L**). The coordinate reference system is OSGB 1936/British National Grid. Land is given by solid grey regions, the survey extent by dashed grey lines. Maps created in QGIS v3.4 (https://qgis.org).

We surveyed the spatial and spectral distribution of ALAN across Plymouth Sound and the Tamar Estuary, UK (50.358° N, 4.169° W) two connected coastal water bodies that are home to the largest naval port in Western Europe and a predominantly LED lit city of more than 240,000 people (Supplementary Fig. S1). Using a combination of radiative transfer modelling and mapping accounting for in situ measured optical seawater properties, we quantified the downwelling irradiance of artificial light at the sea surface [*Ed*(0)], scalar irradiance just below the sea surface [*Eo*(0−)], and scalar irradiance at the seabed as function of tide [*Eo(MHWS)*, *Eo(MLWS)*], depth, sea-surface irradiance, wavelength [blue (400–500 nm), green (495–560 nm), and red (620–740 nm)], and inherent optical sea water properties (Figs. 1, 2). Sea surface spectral irradiances of broadband artificial light were surveyed on four consecutive nights (03/06/2018 to 06/06/2018) during astronomical night when the moon was below the horizon. Cloud conditions (recorded at each station or every 10 min during transit) were variable (0–8 Okta), with observations classified as cloudy (5–8 Okta), or clear (0–3 Okta) for data processing and analysis.

The sea surface of the whole of the lower reaches of the Tamar Estuary and Plymouth Sound (36 km^2^) were exposed to blue, green, and red artificial light during both clear and cloudy conditions (Figs. 1D,H,L, 2D,H,L). Cloudy conditions amplified the sea surface irradiance of blue artificial light by a factor of two on average (mean = 2.12, min = 0.53, max = 4.46), green artificial light by a factor of three (mean = 2.64, min = 0.61, max = 3.83), and red artificial light by a factor of three (mean = 2.75, max = 4.74, min = 0.80) over the survey region. Green artificial light penetrated deepest in the water column compared to the blue and red bands during MHWS in both cloudy (Fig. 1E) and clear (Fig. 2E) conditions (Table 1). Exposure to green artificial light on the seafloor during MHWS under cloudy conditions (5–8 Okta) was three times greater than blue, and seven times greater than red artificial light; and under clear conditions (0–3 Okta) three times greater than blue, and five times greater than red (Table 1). Tidal retreat increased average seafloor exposure to blue, green, and red artificial light by a factor of three (mean = 3.4, min = 1, max = 6.4), two (mean = 2.2, min = 1, max = 3.6), and thirteen (mean = 12.9, min = 1, max = 40.0) respectively during both cloud conditions.

**Table 1:** Exposure to blue, green and red artificial light at night on the seafloor of Plymouth Sound and the Tamar Estuary during clear (0–3 Okta) and cloudy (5–8 Okta) conditions.

Waveband (nm)	Parameter	Cloud cover (Okta)	Irradiance (µW m^−2^)		
			Mean	Min	Max
400–500 (Blue)	*Eo(MHWS)*	0–3	0.89	< 0.1	250.6
495–560 (Green)	*Eo(MHWS)*	0–3	2.6	< 0.1	453
620–740 nm (Red)	*Eo(MHWS)*	0–3	0.4	< 0.1	269.8
400–500 (Blue)	*Eo(MLWS)*	0–3	3.2	< 0.1	606.4
495–560 (Green)	*Eo(MLWS)*	0–3	6.2	< 0.1	872
620-740 nm (Red)	*Eo(MLWS)*	0–3	3.6	< 0.1	903.5
400–500 (Blue)	*Eo(MHWS)*	5–8	1.8	< 0.1	122.6
495–560 (Green)	*Eo(MHWS)*	5–8	5.8	< 0.1	349
620–740 nm (Red)	*Eo(MHWS)*	5–8	1.3	< 0.1	177.7
400–500 (Blue)	*Eo(MLWS)*	5–8	6.1	< 0.1	608.8
495–560 (Green)	*Eo(MLWS)*	5–8	13.6	< 0.1	957
620-740 nm (Red)	*Eo(MLWS)*	5–8	8.8	< 0.1	1,124.9

All values derived from radiative transfer models.

*Eo* scalar irradiance, *MHWS* mean high water spring tide, *MLWS* mean low water spring tide.

We quantified the area of seafloor in the survey region exposed to biologically important blue, green, and red light pollution during each tidal state (MHWS,MLWS), and cloud cover condition (cloudy, clear). To do so, we first defined threshold irradiances in each waveband that could be considered sufficient to stimulate biological responses. Since thresholds of biologically important ALAN are taxon specific and can only be based on the limited number of taxa for which sensitivities have been quantified, a definition of these is inevitably somewhat subjective. If a threshold cannot be representative of all taxa, then it should be precautionary, so that avoiding exceeding it, avoids the majority of impacts. Here, we define ‘biologically important’ artificial light in marine waters as irradiances equal to or greater than the minimum detectable blue (0.19 µW m^−2^), green (0.75 µW m^−2^), and red (150 µW m^−2^) irradiances that elicit diel vertical migration in adult female *Calanus* copepods (such as *C. glacialis, C. finmarchicus*)^7^. *Calanus* copepods are globally widespread, known to be highly sensitive to light^7^ and have been empirically demonstrated to react to sea surface artificial illumination at depths > 80m^1,11^. In addition, the threshold sensitivities of *Calanus* copepods have been quantified separately for blue, green, and red light^7^. Original values provided in Photosynthetic Photon Flux Density (PPFD, µmol photons m^-2^ s^−1^) for wavelengths (λ) of 455 nm (blue), 525 nm (green) and 640 nm (red) were converted to irradiances (µW m^−2^) using [λ x (8.359 × 10^−6^) x PPFD] × 10^6^ where λ is given in metres.

**Figure 3:**
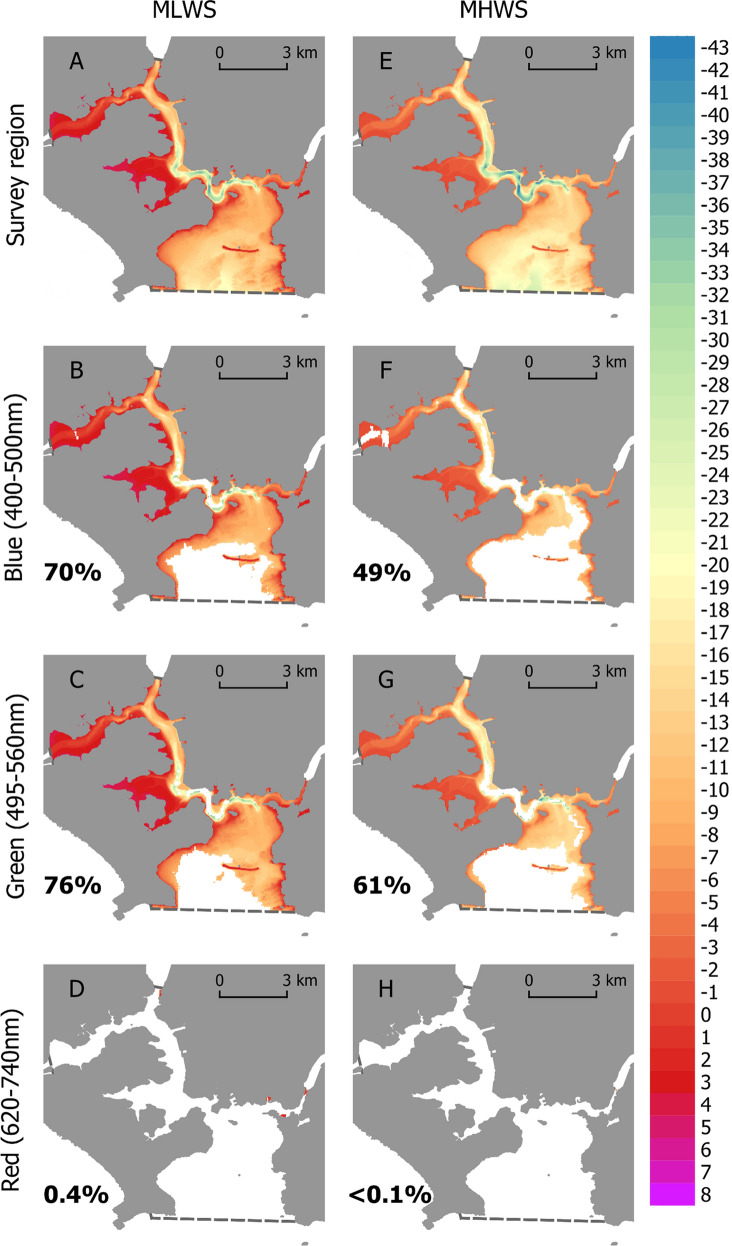
The three dimensional area of seafloor exposed to ‘biologically important’ blue (**B**,**F**), green (**C**,**G**) and red (**D**,**H**) artificial light at night at Mean Low Water Spring (MLWS) and Mean High Water Spring (MHWS) tide in Plymouth Sound and the Tamar Estuary under cloudy conditions. Legend indicates bathymetric depth (m) at the given datums. White space within the survey region (dashed line) indicates exposure at ALAN irradiances below the biological threshold. Numbers inset indicate the percentage of the 3D seafloor region (**A**,**E**) exposed. Maps created in QGIS v3.4 (https://qgis.org).

**Figure 4:**
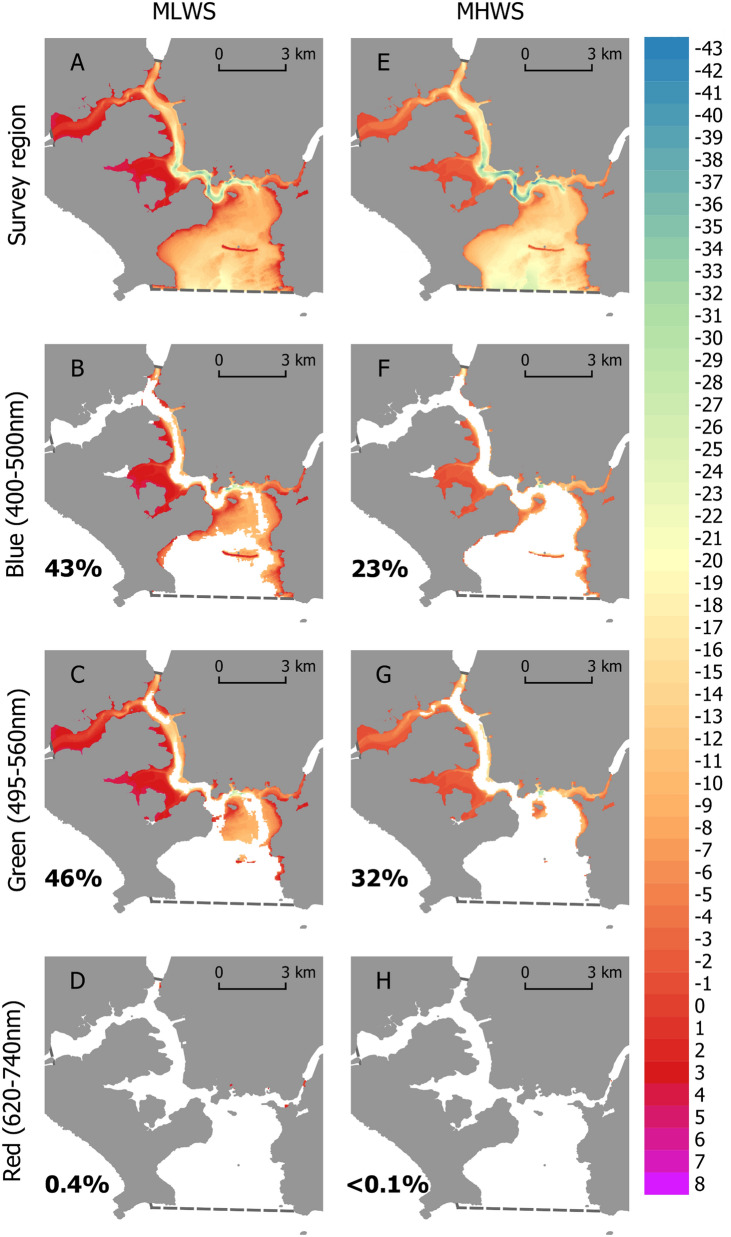
The three dimensional area of seafloor exposed to ‘biologically important’ blue (**B**,**F**), green (**C**,**G**) and red (**D**,**H**) artificial light at night at Mean Low Water Spring (MLWS) and Mean High Water Spring (MHWS) tide in Plymouth Sound and the Tamar Estuary under clear conditions. Legend indicates bathymetric depth (m) at the given datums. White space within the survey region (dashed line) indicates exposure at ALAN irradiances below the biological threshold. Numbers inset indicate the percentage of the 3D seafloor region (**A**,**E**) exposed. Maps created in QGIS v3.4 (https://qgis.org).

We calculated the three-dimensional seafloor area (as opposed to the two dimensional extent) within the survey region exposed to biologically important light pollution in each waveband using the raster surface area calculator in GRASS GIS (Figs. 3, 4). Biologically important green artificial light was most prevalent, with 76% and 46% of the sea floor in the survey region exposed during MLWS tide under cloudy and clear conditions respectively (Figs. 3C, 4C). This area was reduced during MHWS tide to 61% and 32% under cloudy and clear conditions respectively (Figs. 3G, 4G). Biologically important blue artificial light at night was also prevalent, with 70% and 43% of the sea floor in the survey region exposed during MLWS tide under cloudy and clear conditions respectively (Figs. 3B, 4B). This area was also reduced during MHWS tide to 49% and 23% under cloudy and clear conditions respectively (Figs. 3F, 4F). Biologically important red artificial light at night was least prevalent, with 0.4% of the sea floor in the survey region exposed during MLWS tide under both cloud conditions (Figs. 3D, 4D). This area was further reduced during MHWS tide to < 0.1% under both cloud conditions (Figs. 3H, 4H).

## Discussion

Exposure to artificial light at night in marine habitats has been documented in few locations (although see^13^), and the extent to which biologically important artificial light is prevalent on the seafloor has, to our knowledge, not been quantified anywhere in the world. Our results demonstrate that artificial light from coastal urban centres is widespread across the sea surface, sub surface and seafloor of adjacent marine habitats. The areas exposed are non-trivial. Up to 76% of the sea floor in the survey region was exposed to biologically important artificial light. Plymouth is one coastal city with a population of 240,000 people. Given that 75% of the world's megacities (populations > 10 million) are now located in coastal regions^15^ and costal populations are projected to more than double by 2060^6^, it is clear that biologically important light pollution on the seafloor is likely to be globally widespread and increasing in intensity and extent.

Manipulative experiments have already demonstrated that artificially illuminating marine organisms at night to intensities commonly encountered in the real world can alter the structure of marine ecosystems^16,17^, and trophic interactions between marine organisms^18,19^. The physiology, survival, reproduction, and movement of marine fish^20,21^, turtles^22,23^, birds^24^, corals^3^ and other invertebrates^2,25^ are affected by night-time lighting. The documented effects are however, almost exclusively in response to illuminances that would be experienced in close proximity to bright light sources. Our results provide evidence that low sea surface artificial light irradiances caused by sky glow from cities can result in biologically important exposure levels in seafloor habitats. Cloud cover amplifies the propagation of sky glow, a known effect particularly in urban areas^26^ which can disrupt migration undertaken in cloudy conditions in birds^27^ and amphipod crustacean^28^ and is more likely in temperature region/regions further away from the equator. Artificial sky glow extends the geographical influence of localised direct lighting to hundreds of kilometres^29^, suggesting that impacts on marine organisms may be widespread, and urgently need quantifying.

Satellite images have proved valuable for quantifying the exposure of the sea surface and coastal regions to night-time lighting^5,30^, however marine organism life histories play out in pelagic and benthic habitats that experience artificial light undetectable to satellite remote sensing technologies. Using a combination of sea surface mapping and radiative transfer modelling, we were able to produce high resolution (10 m) maps of artificial light on the seafloor that captured: (i) the effect of wavelength and locally important in situ optical water properties on the transmission of artificial light through seawater; (ii) spatially variable seabed bathymetry which affects the path length of artificial light to the seafloor; (iii) temporal variability in this path length due to local tidal conditions; (iv) the natural background irradiance due to stars and the Milky Way; and (v) the influence of cloud cover on the sea surface distribution of artificial light.

We are confident that our estimates are rigorously derived, and do not overestimate exposure to artificial light in the marine habitats of the survey region. They may however, underestimate artificial light exposure for the following reasons, and as such should be considered conservative. Firstly, low irradiances recorded at the furthest extents of the survey region pushed the limits of our radiometers’ sensitivity. Consequently, we may have overestimated the natural background irradiances and in correcting for these, underestimated sea surface irradiances. Secondly, our seafloor irradiances account for the optical properties of a temperate estuarine water body during a snapshot in time, a September 2018 phytoplankton (*Noctiluca scintillans*) bloom. Water column concentrations of optically active constituents (chlorophyll, CDOM, particulates) are strongly seasonal, and even within seasons highly variable meteorological drivers such as precipitation, wind-speed, and temperature, can have a large bearing. The results presented here, although modelled using input measurements representative of those historically observed in this location^31^, may be an underestimate for times of the year where the water column is clearer.

Recent years have seen growing interest in manipulating LED light spectra to avoid wavelengths that give rise to undesirable ecological impacts^32^. Other than a handful of notable examples^33^, evidence of this strategy’s mitigation potential is lacking in marine ecosystems. Our results present a compelling case for using red artificial light at night in coastal installations to reduce exposure in marine habitats. Red light attenuates faster in water and is less visible to marine animals. Given this, it is unsurprising that 0.4% of the seafloor was exposed to biologically important red artificial light, compared to up to 70% and 76% blue and green artificial light respectively. The persistence of these attenuation differences in geological time means that many marine animals have also evolved maximum sensitivity at shorter wavelengths of their visual spectrum compared to terrestrial^14^. While the ecological benefits of applying spectral manipulation in terrestrial ecosystems remain uncertain^11,32^ , its application in aquatic habitats seems likely to produce favourable results. Nonetheless, red and amber light spectra guide developmental, behavioural, and physiological processes in a number of marine organisms^34,35^, and it seems unlikely that switching to long wavelength emitting light sources or retrofitting existing luminaires with band pass filters will avoid the ecological impacts of artificial light altogether. The feasibility of adopting such approaches is also likely to prove highly contentious in maritime industries and communities. Alternative strategies including switching lights off, dimming or shielding lights, and preserving naturally dark seascapes should be given equal consideration in the design of coastal lighting installations^11^.

The rapid urbanisation of global coastlines is increasing the exposure of marine waters to artificial light, including those regions of our oceans most valued by society^36^. We conclude that artificial light pollution originating from a coastal city is sufficient to cause widespread exposure of adjacent seafloor habitats to biologically important light pollution. The transition of outdoor lighting to technologies rich in short wavelength light will exacerbate exposure levels on the seafloor. We suggest that LEDs and other broad spectrum light sources should be considered emerging threats to marine biodiversity that warrant urgent attention.

## Materials and methods

### Surveying ALAN

GPS and time stamped irradiances were measured every 10 s from on board the R.V. Plymouth Explorer along a continuous transect linking 90 pre-allocated sampling stations (Supplementary Fig. S1) using a Spectrosense 2 + data logger fitted with a multispectral irradiance sensor (Skye Instruments Ltd). Each measurement was recorded from 1 m above the sea surface to avoid detecting upwardly emitted light from the port and starboard navigation lights which remained on to ensure the vessel was visible at night in close proximity to a busy military port.

The inherent optical properties (IOPs) of the water column (absorption and scattering of light by, for example algae, sediments and coloured dissolved organic matter) play a critical role in determining the propagation of artificial light in seawater. We accounted for them by quantifying the sub-surface in-water optical properties (IOPs) at 43 station locations using a pole mounted Wet Labs BBFL2 which measures backscatter (b_b_) at 532 nm, chlorophyll fluorescence calibrated as chlorophyll concentration (mg m^−3^) and fluorescence due to coloured dissolved organic matter (fCDOM). The instrument was held just below the surface for a period of 3 min, using a 1 Hz sampling rate, to enable a representative amount of data (*n* > 180) to be collected.

### Mapping sea surface irradiances

All spatial data manipulation was carried out in QGIS version 3.4. Data processing was carried out separately for data collected under cloudy and clear conditions such that the influence of cloud on the extent and intensity of artificial light could be established. Continuous recordings of artificial light irradiances logged during transit at variable speeds resulted in highly uneven sample densities. To remove the leverage of densely sampled regions, data were first resampled across a 100 m resolution, 100 m diameter circular buffered grid overlaid across the survey region. The median blue, green, and red irradiance values falling within each buffered grid point were then extracted, and interpolated to 10 m resolution sea surface irradiance maps by kriging using an exponential semi-variogram model.

### Correcting for background sky brightness

The resulting rasters were corrected for natural background irradiance using the intercept of the relationships between interpolated broadband blue, green, and red irradiances and predicted night sky brightness (mCd m^−2^) from the New World Atlas of Night Sky Brightness^29,37^ which were extracted for pre-allocated sampling stations for both x and y data (Supplementary Fig. S2). These relationships were quantified using quantile regression on the median to reduce the leverage of irradiances recorded under direct light sources including bridge and port lights (Supplementary Fig. S2). The night sky brightness data were supplied corrected for natural background lighting from the stars and Milky Way, hence the intercept of these relationships can be taken as irradiance in the absence of artificial light and was subtracted from the surface irradiance maps to correct for natural background light sources.

### Seafloor bathymetry

A 10 m resolution Mean Sea Level (MSL) bathymetry map for the region was obtained from the Channel Coastal Observatory (www.channelcoast.org) datasets and converted to depth at Mean High Water Spring (MHWS) and Mean Low Water Spring (MLWS) tide using the local Vertical Offshore Reference Frames.

### Modelling ALAN propagation in seawater

Sea surface broadband blue, green, and red irradiances under cloudy and clear conditions, and depths at MHWS and MLWS were extracted across a 50 m resolution point grid to define the input parameters of a hydrological optics model [HYROLIGHT^38^] of downwelling irradiance at the sea surface [*Ed*(0)], scalar irradiance just below the sea surface [*Eo*(0−)], and scalar irradiance at the seabed as a function of tide [*Eo(MHWS)*, *Eo(MLWS)*], depth, sea-surface irradiance, wavelength, and inherent optical sea water properties. For areas of seabed exposed during low tide (MLWS), the value of *Eo(MLWS)* was set to *Ed*(0). The measured IOPs were interpolated onto the same 10 m resolution grid and extracted in the same way as for the sea surface irradiances, and used to model optical properties of the water column (Supplementary Fig. S2).

For *Ed*(0), the broadband surface spectral irradiances were defined as blue (400–500 nm), green (495–560 nm), and red (620–740 nm). The sky radiance distribution was modelled as uniform for both cases of cloud cover conditions (i.e. *Ed*(0) is totally diffused). For the in-water radiance distribution [*Eo*(0−), *Eo(MHWS)*, *Eo(MLWS)*], four inherent optical property (IOP) components were included in the model, these being pure-water, chlorophyll, CDOM, and particulates. Spectral absorption and scatter for pure water was taken from^39^ and used a pure water phase function^40^ for the angular light distribution. Spectral absorption due to chlorophyll was modelled using chlorophyll concentration^41^, and was assumed to be non-scattering. CDOM spectral absorption was determined using the CDOM fluorescence measurements together with the approach of^42^ and assumed to be non-scattering. Spectral particulate backscattering was derived from the b_b_ (532 nm) interpolated field measurements using a power law relationship^43^ and the phase function of^44^. The resulting 10 m resolution map resulted in 22,231 discrete points with inputs of above surface irradiance (3 wavelengths) and in-water IOPs to be run at two cloud cover conditions. Each discrete point also had two extracted water column depths of the height of MHWS and MLWS, resulting in three depths the HYDROLIGHT model was run at: sub-surface (0.01 m), MHWS, and MLWS tide depth. The HYDROLIGHT model was run on a Linux workstation for a total of 133,386 cases (total run time 10 h), and values of the scalar irradiances in blue, green and red spectrum were extracted at these three depths for each of the discrete points.

## Supplementary information

Supplementary figures.
