# Supplementary material for: Biologically important artificial light at night on the seafloor
Source: Sci Rep. 2020 Jul 27;10:12545. doi: 10.1038/s41598-020-69461-6 (PMC7385152; doi:10.1038/s41598-020-69461-6)
Supplement: Supplementary file 1 — Supplementary figures. [file 41598_2020_69461_MOESM1_ESM.pdf]

Biologically important artificial light at night on the seafloor

Thomas W. Davies[1,2*], David McKee[3], James Fishwick[4], Svenja Tidau[1,2] and Tim Smyth[4]

Supplementary Information

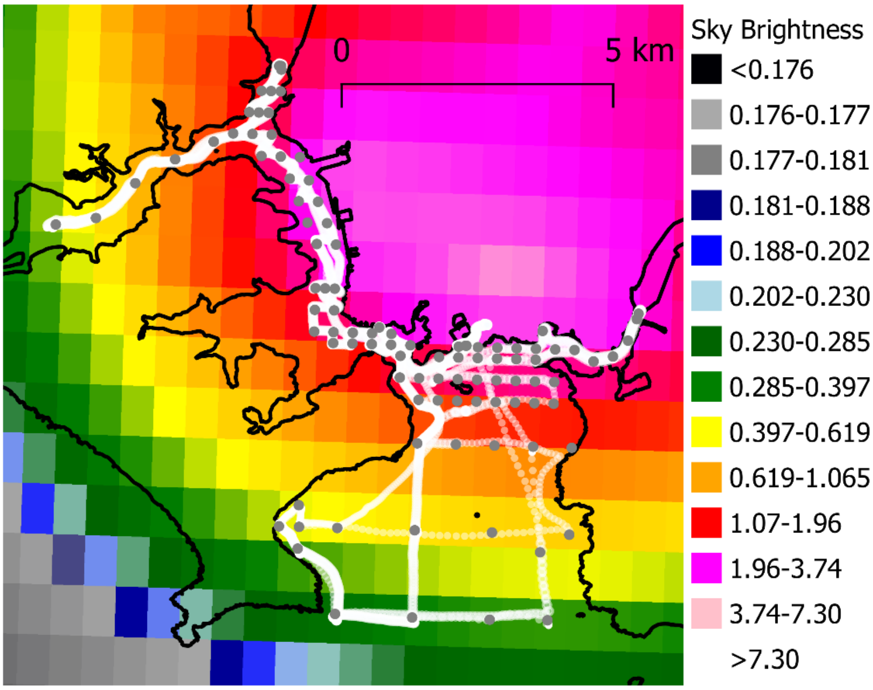

Figure S1. Artificial sky brightness (mCd m^{-2}) predicted across Plymouth Sound and the Tamar Estuary by Falchi *et al.*[25,33]. Coastline in black is given at Mean High Water Spring tide. Pre-allocated sampling stations are given by open points. The multispectral irradiance distributions of ALAN were recorded in continuous tracks between survey stations (transparent points).

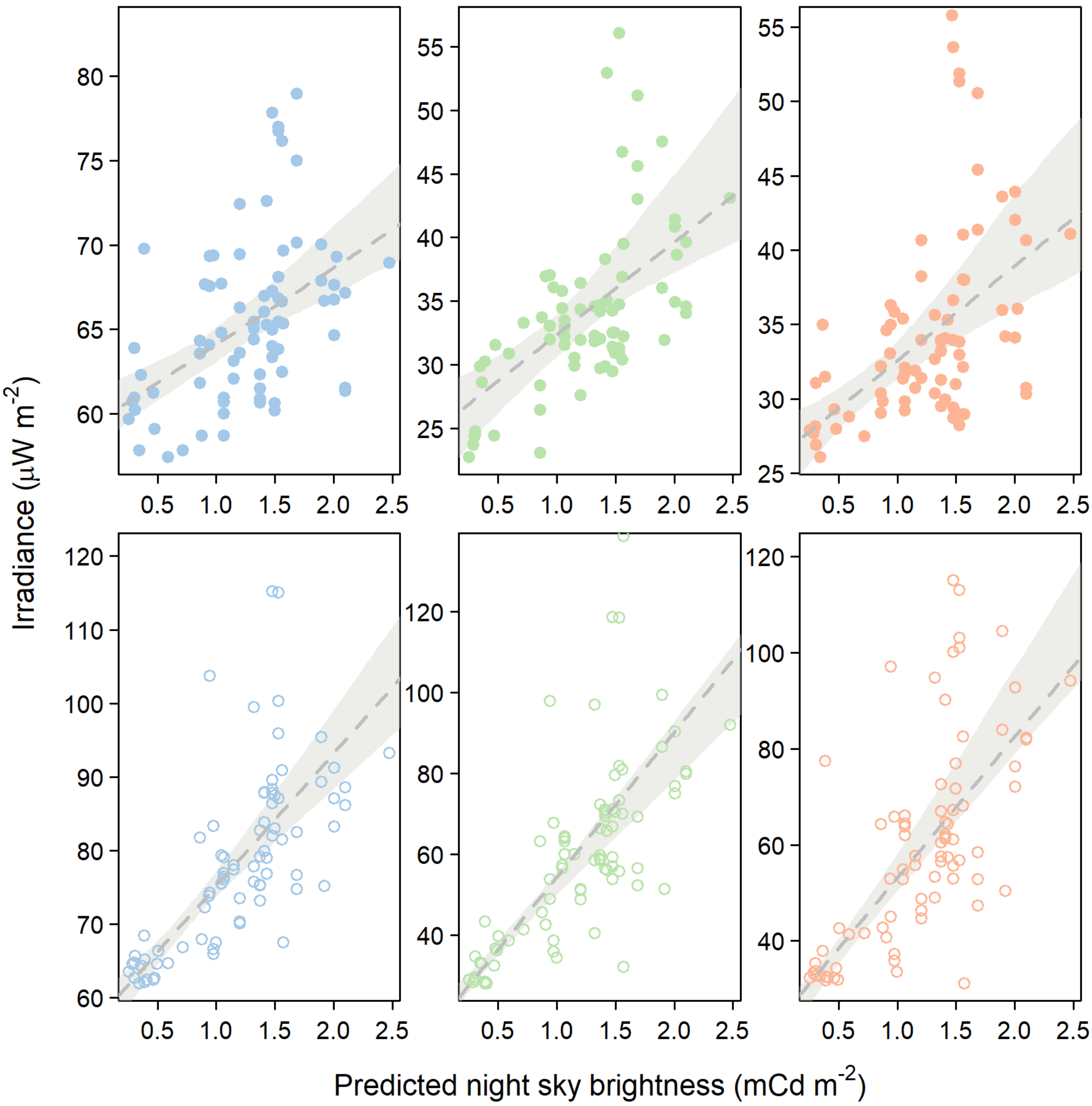

Figure S2. The relationships between measured blue (400-500nm), green (495-560nm) and red (620-740nm) sea surface irradiance and predicted night time sky brightness during a new moon clear night [from the New World Atlas of Artificial Night Sky Brightness; Falchi *et al.*[25,33]]. Relationships (dashed line) are fitted using quantile regression on the median to reduce the leverage of measurements taken directly under artificial light sources, which are not representative of sky brightness. Closed and open points denote data recorded on low cloud (0-3 Okta's) and high cloud (5-8 Okta's) nights respectively.
